# Closed Endotracheal Suction Systems for COVID-19: Rapid Review

**DOI:** 10.2196/42549

**Published:** 2023-01-10

**Authors:** Carmen Amaia Ramírez-Torres, Félix Rivera-Sanz, Teresa Sufrate-Sorzano, Azucena Pedraz-Marcos, Ivan Santolalla-Arnedo

**Affiliations:** 1 Health and Care Research Group University of La Rioja Logroño Spain; 2 Research Unit on Health System Sustainability Biomedical Center of La Rioja Logroño Spain; 3 Unit of Data Biomedical Center of La Rioja Logroño Spain; 4 Department of Nursing University Autonoma of Madrid Madrid Spain

**Keywords:** endotracheal suctioning, closed suction system, rapid review, suction, mechanical ventilation, COVID-19, intensive care unit, health intervention, endotracheal, patient care, healthcare, ventilator, health benefit

## Abstract

**Background:**

The increase in admissions to intensive care units (ICUs) in 2020 and the morbidity and mortality associated with SARS-CoV-2 infection pose a challenge to the analysis of evidence of health interventions carried out in ICUs. One of the most common interventions in patients infected with the virus and admitted to ICUs is endotracheal aspiration. Endotracheal suctioning has also been considered one of the most contaminating interventions.

**Objective:**

This review aims to analyze the benefits and risks of endotracheal suctioning using closed suction systems (CSS) in COVID-19 patients.

**Methods:**

A rapid review was carried out using the following databases: PubMed, MEDLINE, CINAHL, LILACS, the Cochrane Library, and IBECS. The data search included articles in English and Spanish, published between 2010 and 2020, concerning adult patients, and using the key words “endotracheal,” “suction,” and “closed system.”

**Results:**

A total of 15 articles were included. The benefits and risks were divided into 3 categories: patient, care, and organization. Relating to the patient, we found differences in cardiorespiratory variables and changes in the ventilator, for example, improvement in patients with elevated positive and end-expiratory pressure due to maladaptation and alveolar collapse. Relating to care, we found a shorter suctioning time, by up to 1 minute. Relating to organization, we found fewer microorganisms on staff gloves. Other conflicting results between studies were related to ventilator-associated pneumonia, bacterial colonization, or mortality.

**Conclusions:**

Aside from the need for quality research comparing open suction systems and CSS as used to treat COVID-19 patients, closed endotracheal suctioning has benefits in terms of shorter stay in the ICU and reduced environmental contamination, preventing ventilator disconnection from the patient, reducing the suctioning time—though it does produce the greatest number of mucosal occlusions—and preventing interpatient and patient-staff environmental contamination. New evidence in the context of the SARS-CoV-2 virus is required in order to compare results and establish new guidelines.

## Introduction

The effects of a disease such as COVID-19 have a global reach and can be a severe hindrance to society. Among patients diagnosed with COVID-19, 5% require admission to an intensive care unit (ICU), and, of these, 88% require mechanical ventilation (MV) to support their breathing [1].

COVID-19 is caused by the SARS-CoV-2 virus, transmitted by aerosols. During ICU admission, the patient is in the symptomatic phase of the disease, with a significant viral load, and can pose a significant health risk due to this airborne transmission, particularly to health care professionals because of the type of procedures used for patient stabilization and clinical recovery [2].

Understanding the infection mechanisms of SARS-CoV-2 requires studies of the procedures and interventions that cause greater risk of aerosol expansion. A systematic review by Jackson et al [2] found 14 procedures that are widely recognized as important generators of aerosols, including, most importantly, intubation and extubation, suction of the airways, bronchoscopy, and noninvasive ventilation.

Endotracheal suctioning is one of the most common procedures in patients intubated in an ICU. The intervention requires essential care in the form of oxygenation before suctioning, at the time of suction, and after suctioning. These procedures are performed by nurses. Endotracheal suctioning requires specialized staff, as, though it is a common procedure, it can occasionally cause harm to the patient. The types of harm directly associated with endotracheal suctioning include 6 that are particularly important when managing critical patients: asynchrony with the ventilator, hypoxia, hemodynamic alterations, atelectasis, pain, and damage to the tracheal mucosa [3-5].

There are 2 different systems for performing endotracheal suctioning, the more common open suction system (OSS) and the closed suction system (CSS). There are currently arguments for and against both systems [3].

CSS prevent the diffusion of aerosols in the outside air, thus reducing the risk of contamination for hospital staff in terms of environmental pollution. Although theoretically, it seems the best option, no national nor international studies have yet been published that evaluate the benefits and risks for patients with COVID-19 [2].

The objective of this review was to analyze the benefits and risks of closed endotracheal suctioning. The specific objectives were to (1) describe the benefits and risks of CSS with respect to OSS in patients connected to a mechanical ventilator and (2) evaluate which benefits are useful for the treatment of COVID-19 patients connected to a mechanical ventilator.

## Methods

A narrative rapid review was carried out according to the Cochrane Rapid Reviews Method Group criteria, which define a rapid review as “a form of knowledge synthesis that accelerates the process of conducting a traditional systematic review through streamlining or omitting specific methods to produce evidence for stakeholders in a resource-efficient manner” [6].

### Setting the Research Question—Topic Refinement

The formulation of the question followed the objectives of the review [7].

The MeSH term used for the search strategy was “endotracheal suction.” To carry out a more advanced search, we added the term “closed system.“

### Setting the Eligibility Criteria

We included articles following the inclusion and exclusion criteria (Textbox 1). We considered all literature containing the keywords, as long as the article contained information about CSS in adult patients.

The systematic search was carried out between November 1, 2020, and December 30, 2020.

Although the criteria indicate articles from 2010 onwards, we did include previous articles that we considered to contain essential information for our analysis.

**Summary of the inclusion and exclusion criteria.**

**Inclusion criteria**

- Published between 2010 and 2020
- Published in English or Spanish
- Included a closed suction system
- Included intensive care unit (ICU) patients

**Exclusion criteria**

- Published prior to 2000
- Not a pediatric study

### Search Procedure

#### Search Strategy 

An electronic bibliographic search was carried out using the following databases: PubMed, MEDLINE, CINAHL, LILACS, the Cochrane Library, and IBECS. Some relevant articles were also selected from the bibliographic references of the articles found through the systematic search.

The selected keywords were “closed endotracheal suction system” and “COVID-19.”

#### Data Collection

We first followed the PRISMA (Preferred Reporting Items for Systematic Reviews and Meta-Analysis) process for data extraction [8]. The articles were first selected by title; we then narrowed the selection by reading abstracts and finally by reading the texts in full, dividing the work among researchers in the group. Of the 157 articles selected, after eliminating duplicates and applying the inclusion and exclusion criteria, we were left with 25 articles to read in full.

The 25 articles were read by 2 researchers, considering the objective and design of the study, and 2 other researchers selected those they considered suitable for the results. After this selection, the researchers read the articles and noted the most relevant aspects, establishing categories for the benefits and risks found. Finally, the researchers focused on the quantitative and qualitative nature of the results found.

## Results

### Articles

**Figure 1:**
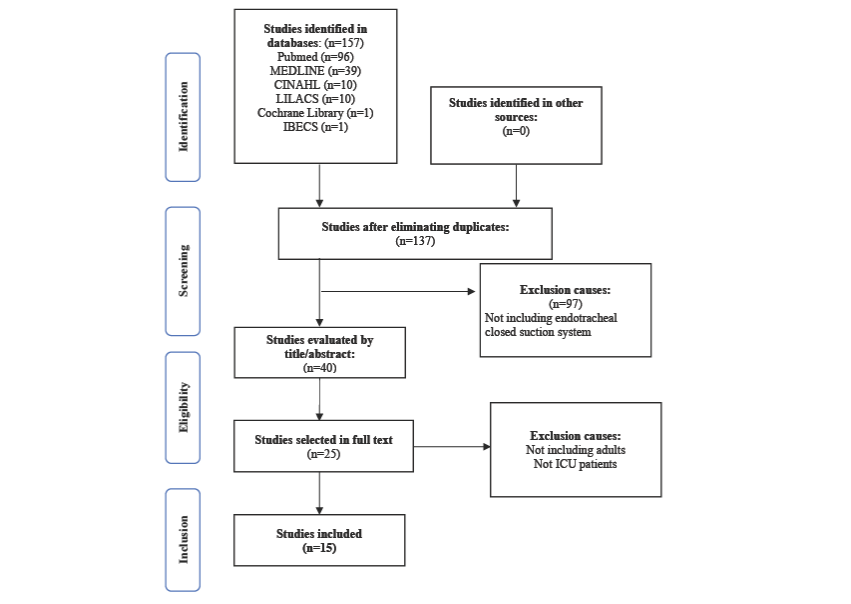
PRISMA (Preferred Reporting Items for Systematic Reviews and Meta-Analyses) diagram flow. ICU: intensive care unit.

At the end of the selection process, we included 15 articles (Figure 1). It is important to highlight that they varied in terms of their methodology, from meta-analysis to randomized clinical trials and observational studies. All the articles met the criteria outlined in the methodology, using 1 reviewer to examine the final selection of articles and another reviewer to read the excluded articles in full.

The articles included were published between 2003 and 2020 (Table 1). All the included articles that were dated before 2010 were considered of special relevance and found through the search articles. Of the 15 articles, 9 were developed in collaboration with or within the European Union. The studies varied in design: 1 in vitro trial, 2 meta-analyses, 2 reviews, 2 quasi-experimental studies, 3 clinical trials with small patient samples, and 5 observational studies. 

**Table 1:** Details of the included articles.

Article	Aim	Design	Participants
Jongerden et al (2007) [9]	To review the effectiveness of CSS^a^ and OSS^b^ in terms of cross contamination and economic cost	Meta-analysis	15 randomized clinical trials
Subirana et al (2007) [10]	To compare the use of CSS and OSS in patients on ventilators for more than 24 hours	Review	16 trials (1684 patients)
Elmansoury and Said (2017) [11]	To compare the use of CSS and OSS in patients on ventilators for more than 24 hours	Randomized clinical trial	141 patients
Siempos et al (2008) [12]	To evaluate if CSS prevent VAP^c^	Meta-analysis	9 randomized clinical trials
Zeitoun et al (2003) [13]	To verify the incidence of VAP with the use of CSS	Clinical trial	47 patients
Dave et al (2011) [14]	To evaluate the effectiveness of tracheal suctioning with a CSS	In vitro model	—^d^
Faradita Aryani and Tanner (2018) [15]	To compare the use of CSS and OSS in ventilated ICU^e^ patients	Systematic review	5 studies
Jongerden et al (2012) [16]	To assess changes in heart rate, average arterial blood pressure, and peripheral oxygen saturation after endotracheal suctioning with a CSS	Randomized prospective observations	197 observations of endotracheal suctioning
Åkerman et al (2014) [17]	To compare the use of CSS with OSS in cases of VAP, bacterial contamination, and adverse circumstances	Observational cohorts	126 patients
Özden and Görgülü (2015) [18]	To compare the effects on the hemodynamics of patients undergoing open heart surgery	Quasi-experimental	120 patients
Adi et al (2013) [19]	To evaluate ETT^f^ compared with new or unused ETTs in terms of changes in inspiratory resistance or peak inspiratory pressure	Observational	16 ETTs
de Fraga Gomes Martins et al (2019) [20]	To compare the suction volume, respiratory mechanics, and hemodynamics of patients treated with OSS/CSS and with inspiratory pause	Randomized clinical trial	31 patients
De Seta et al (2020) [21]	To establish a step-by-step protocol for patients with tracheotomy who require MV^g^	Observational	15 patients
Ricard et al (2011) [22]	To compare the contamination of gloves and the airway while using OSS and CSS	Quasi-experimental	19 cases of endotracheal suctioning
Vargas and Servillo (2020) [23]	To evaluate the use of an improvised CSS in a case of COVID-19	Observational	12 patients

^a^CSS: closed suction systems.

^b^OSS: open suction systems.

^c^VAP: ventilator-associated pneumonia.

^d^Not applicable.

^e^ICU: intensive care unit.

^f^ETT: endotracheal tube.

^g^MV: mechanical ventilation.

For all of the articles included, we identified the benefits and risks of CSS and OSS and classified them according to whether they were primary or secondary outcomes. A summary of the findings related to patient, care, and organization can be found in Table 2. 

**Table 2:** Summary of the results of closed suction systems versus open suction systems.

Outcomes			Closed suction system		Open suction system
**Patient-related outcomes**					
	VAP^a^	No differences [11]		Nonsignificant reduction in studies with small samples [24]; increases risk by facilitating microaspiration from the upper to the lower section [12]	
	Mortality	No differences [11]		—^b^	
	Cardiorespiratory variables	No differences in HR^c^, ABP^d^, and SpO_2_^e^ [16]		HR and ABP slightly more stable [18]; better SpO_2_ recovery after pre-oxygenation [18]; better ABP and hypoxemia during heart surgery [18]	
	Bacterial colonization	No differences in the most common microorganisms [17]		Increased colonization (*Pseudomona aeuroginosa*, *Escherichia coli*, *Staphylococcus aereus*, and *Acinetobacter spp)* [9-11,21]	
**Care-related outcomes**					
	Changes to the ventilator	No significant differences relating to PEEP^f^ [12]; less time spent connected to MV^g^ [11]		Better Ri^h^ and PIP^i^ after first suctioning [19]; improvement in patients with elevated PEEP due to maladaptation and alveolar collapse [16]	
	Nursing care–related	No differences in 10-second to 15-second suctions [18]; more effective in removing secretions [16]		Variable number of suctions: every 3 hours or the minimum possible [18]; improvement in vital constants with pre-oxygenation [16]; expiratory pause, increased volume of suctioned secretions [20]; shortest suctioning time, up to 1 minute less [24]; increased number of ETT^j^ occlusions requiring replacement and obstructions [17]	
**Organization-related outcomes**					
	Cost-effectiveness	Less cost- effective [9,11,16]		Less use of gloves, masks, and glasses [9]; greater cost or prolonged use (>72 hours) [11,24]	
	Environmental or cross contamination	No differences [9,17]		Fewer microorganisms found on staff gloves [21]	
	Time spent in the intensive care unit	No differences [11]		—	

^a^VAP: ventilator-associated pneumonia.

^b^Not applicable.

^c^HR: heart rate.

^d^ABP: arterial blood pressure.

^e^SpO_2_: peripheral oxygen saturation.

^f^PEEP: positive and end-expiratory pressure.

^g^MV: mechanical ventilation.

^h^Ri: inspiratory resistance.

^i^PIP: peak inspiratory pressure.

^j^ETT: endotracheal tube.

### Primary Outcomes

The baseline condition of the patients or their pathologies was the variable that most influenced the analysis of the benefits and risks of endotracheal suctioning [10].

#### Ventilator-Associated Pneumonia

Elmansoury and Said [11] carried out an analysis in 2 groups: 1 intervention group (n=66) with a CSS and 1 control group (n=75) with an OSS for 6 months with the possible incidence of ventilator-associated pneumonia (VAP). They found no statistically significant differences: 30.13 VAP per 1000 ventilator days in the control group and 17.48 VAP per 1000 ventilator days in the intervention group. Jongerden et al [9] and Subirana et al [10] found no statistically significant differences between VAP with CSS and VAP with OSS (odds ratio [OR]=0.96, 95% CI 0.76-1.21; n=1377, risk ratio [RR]=0.88, 95% CI 0.70-1.12). A slight reduction was found when using a CSS in studies with a small sample size (n=9); for example, Zeitoun et al [13] found no significant differences, but the frequency of VAP in cases treated with OSS was 11 of 24 cases, while the frequency of VAP in cases treated with CSS was 10-14 of 23 cases. However, in the study by Dave et al [14], the researchers concluded that CSS facilitate the microaspiration of fluid from the upper zone to the lower zone, increasing the risk of VAP. In addition, the variation in VAP definition criteria and the absence of a clear description make adequate comparison impossible. Siempos et al [12] and Jongerden et al [9] included articles that defined VAP using more quantitative or qualitative results, for example different temperatures, time with MV, or colony-forming unit [9,12,13].

#### Mortality

No significant differences in mortality were found in any of the included studies. For example, Jongerden et al [9] and Subirana et al [10] found no statistically significant differences between CSS and OSS (OR=1.02, 95% CI 0.84-1.25 and RR=1.02, 95% CI 0.84-1.23, respectively) [10,11,24]. The systematic review by Faradita Aryani and Tanner [15] included 435 prospective studies, concluding that none of the studies found differences regarding increased VAP or resultant mortality.

### Secondary Outcomes 

#### Cardiorespiratory Variables 

In only 1 of the studies, the variables were more stable with the use of closed systems. Even so, heart rate (HR) was almost imperceptible, and there were no significant differences in arterial blood pressure (ABP) [10,13]. Jongerden et al [16], with a total of 165 patients using CSS and OSS, measured physiological parameters including HR, ABP, and peripheral oxygen saturation (SpO_2_), without noting any differences. In this study, they found notable—although not significant—differences in SpO_2_ recovery after pre-oxygenation in patients using CSS (96%-99%) and OSS (95%-98%) [10]. Likewise, in terms of oxygen saturation, no differences were found in the study carried out by Åkerman et al [17]. However, Özden and Görgülü [18] concluded that HR with OSS increased at 5 minutes and 15 minutes after the procedure and hypoxemia can in fact be avoided using a CSS while also improving ABP in postoperative patients, particularly after heart surgery.

#### Relating to Changes to the Ventilator 

Adi et al [19] examined aspects relating to the ventilator, such as inspiratory resistance (Ri) and peak inspiratory pressure (PIP); they estimated endotracheal tube (ETT) obstruction at extubation, taking into account patients with more than 12 hours of MV and obtaining an improvement in both Ri and PIP after the first suction with a CSS. Dave et al [14], using a simulation model without patients, concluded that using a CSS does not achieve positive results in terms of maintaining positive and end-expiratory pressure (PEEP). de Fraga Gomes Martins et al [20] and Jongerden et al [16] did not achieve the same results, but both did recommend the use of CSS in patients who require elevated PEEP to prevent alveolar collapse in order to avoid asynchrony to MV. There were no significant differences in the time ICU patients were connected to MV (weighted mean difference [WMD]=0.44, 95% CI 0.92-1.80) [10]. Subirana et al [10] mentioned the time patients were connected to MV only, without conducting an analysis. However, Siempos et al [12] mentioned CSS was associated with longer MV duration (WMD=0.65 days, 95% CI 0.28-1.03) [9].

#### Bacterial Colonization

Some studies demonstrated an increase in colonization while using a CSS, with a 49% increased risk in comparison with OSS (OR=2.88, 95% CI 1.52-5.52) [9,10,13]. Åkerman et al [17], in their cohort study, included 126 patients: 61 using an OSS and 65 using a CSS. Both groups showed colonization with similar gram-negative bacteria, the most common being *Pseudomona aeuroginosa*, *Escherichia coli*, and *Staphylococcus aereus*; other studies connected colonization to *Acinetobacter spp.* without general VAP-related differences. However, Elmansoury and Said [11] found greater incidence of *Acinetobacter spp.* and *Pseudomonas aeuroginosa* (causative of VAP) with CSS as well as no incidence of methicillin-resistant *Staphylococcus aureus* and *Staphylococcus aereus.*

#### Nursing Care-Related

##### Suctioning Techniques

The lack of description of suctioning techniques and their characteristics in the articles makes comparison impossible [10,13].

The application of oxygenation prior to endotracheal suctioning is an important variable. For example, Jongerden et al [16] found differences in SpO_2_ recovery when pre-oxygenation was used with a CSS. Özden and Görgülü [18] described the suctioning protocol: 1 minute of pre-oxygenation at 100%, universal precautions, for 10 seconds to 15 seconds, using the smallest possible level of suction (<120 mm Hg), and oxygenation at 100% for another minute. In the randomized clinical trial by de Fraga Gomes Martins et al [20], they provided a detailed description of the process: pre-oxygen at 100% for 1 minute before suctioning 3 times for 10 seconds. This procedure was followed for the control group, while an expiratory pause was included for the intervention group. The authors concluded that the expiratory pause resulted in an increase in the volume of secretions suctioned [20].

##### Suctioning System 

The type of device used in CSS was only described in 1 of the articles, and we decided to discard studies on single-use devices because this means disconnecting the patient from the ventilator [11,19].

##### Suctioning Time and Frequency

Suctioning time is a necessary variable because it has an impact on workload and it can alter hemodynamics for more or less time [10]. The 2012 observational study by Jongerden et al [16] found no significant differences between one system and another; the suctioning processes lasted 10 seconds to 15 seconds and had the same effect on the vital constants. Work overload is another aspect that must be taken into account. It has been observed that using a CSS can be up to 1 minute faster: 2.5 minutes for OSS in comparison with 1.5 minutes for CSS [10]. 

In terms of the number of suctions, differences in recommendations range from performing endotracheal suctioning every 3 hours, to a minimal number and only when strictly necessary [22]. Frequent suctions can provoke hemodynamic instability, damage to the tracheal mucosa, hemorrhage, and infection [19]. 

##### Suctioning Results 

The quantity of suctioned secretions has not been studied in depth despite the importance of these results [10,20]. OSS are considered more effective in removing secretions, but the articles only discussed experiments in vitro or in animal models [16]. In the study by Åkerman et al [17], 3 ETT occlusions and 3 severe obstructions were reported in patients using an OSS, while in the CSS group, only 1 occlusion was reported.

#### Organization-Related

##### Economic Benefit

Jongerden et al [9] found that the cost of CSS is between 14 and 100 times the cost of OSS, but there is less need for personal protective equipment (gloves, masks, and glasses) when using CSS.

There is some debate on this issue, although most studies associate higher costs with the use of closed systems, with the exception of systems connected for at least 72 hours (24 hours is recommended) [9,10]. However, prolonged use has the disadvantage of increasing bacterial colonization [9,10].

##### Environmental or Cross Contamination

The fewer the disconnections, the less likely that pathogens will be spread into the environment. No differences in transmissions were found between patients in the same place and receiving care from the same staff [9,17]. However, Ricard et al [22] highlighted that there was in fact less risk of glove contamination with microorganisms using a CSS.

##### Time Spent in the ICU

No differences were observed in relation to time spent in the ICU [10]. The 2007 meta-analysis by Jongerden et al [9] included 15 clinical trials using CSS and OSS and concluded that none of the benefits associated with CSSs are scientifically proven.

### COVID-19

It is important to set out the benefits of a CSS in order to manage the benefits and risks faced by COVID-19 patients.

The protocol followed by De Seta et al [21] in COVID-19 patients with tracheotomy recommended mucus management using 3 elements: humidifiers, bacterial and viral filters, and CSS. These 3 elements are intended to prevent the spread of SARS-CoV-2 viral aerosols [21].

In the article by Vargas and Servillo [23], we found an alternative to CSS that arose from the shortage of CSS during the global pandemic. This system is composed of an OSS with the addition of the sterile sheath that is commonly used when performing ultrasound scans. However, there are no results on the effectiveness of CSS in COVID-19 patients [23].

## Discussion

### Principal Findings

This rapid review established 3 different categories for comparing the benefits and risks of the use of CSS versus OSS. The review compared 11 different outcomes. Using this classification, we can establish comparability indicators for future studies despite the fact that the indicators described are not significantly conclusive. Consideration should be given to the proposed benefits, which could make a significant difference to the procedures for treating COVID-19. 

As seen in the Results section, there is ample variability in the conclusions of the studies. After analysis, we concluded that there is a need for the development and implementation of clinical practice guidelines on suctioning. This is because, in all cases, the implementation of suctioning guidelines that include a protocol for the technique improves outcomes for patients [25].

Among the benefits of CSS, the most discussed is the benefit and risk with respect to VAP, referred to in al the included articles. Although there was no significant evidence in most of the studies that CSS protect against VAP, all possible measures should be taken to avoid co-infection, as this leads to increased morbidity and mortality [26]. The fact that CSS can increase the risk of VAP should provide motivation for further studies that take into account actions to prevent this increase in colonizations, such as aspiration of subglottic secretions or oral hygiene.

One of the most positive aspects of CSSs is the avoidance of asynchrony and discomfort, maintaining PEEP and avoiding hypoxemia during suctioning [27,28].

This is because patients with COVID-19 require close ventilatory support [29]. 

However, it is important to highlight the key risks of CSS in order to apply possible measures of prevention. ETT obstruction is a proven complication as CSS fail to suction the same amount of sputum as OSS [9]. This is especially important with COVID-19 since obstruction of an ETT requires new intubation or the application of the Ambu ventilation procedure, which increases the risk of generating aerosols [2,30,31]. 

The economic cost of CSS is higher than that of OSS, although studies have shown that extended use of CSS can improve the cost-effectiveness. However, CSS are increasingly popular, and the benefits include reduced time spent performing tracheal suction, which frees nurses up for other important activities. We must bear in mind that there are different types of CSS, health care staff require training to use them, and protocols for correct use must be established by the unit or the manufacturer [10,32]. 

Another important benefit is the reduction of cross contamination or infection of the staff themselves. According to the World Health Organization [33], around 14% of infections worldwide occur among health care staff, which, in consideration of possible future virus outbreaks, is further incentive to improve their protection through the use of resources such as CSS [34].

There are differences in the samples obtained from contaminated gloves worn by health care staff using suction systems: 9 of 9 gloves were contaminated during tracheal suction using OSS, and 3 of 10 gloves when using CSS [22]. For COVID-19, contamination depends on the stage of infection as well as the interventions performed, with suctioning standing out as one of the most infectious interventions. Reducing the exposure time of staff each time a suctioning procedure is performed can be another major benefit [34].

### Limitations

The major limitation of this review is the methodological quality of the studies included and the inability to carry out reliable comparisons. Most of the studies applied their selection criteria on the basis of convenience and inaccurately described the intervention carried out.

In addition, there are limitations relating to language and document access, as only fully accessible documents were included. The majority of study samples are small. It should be noted that fighting the COVID-19 global pandemic has required huge economic and human resources, which has reduced the resources applied to research and the production of specific literature on the subject.

### Comparison With Prior Work

Despite these limitations, the strengths of the review include it serving as a starting point for future research and the fact that it was carried out at the time of the pandemic and includes quality and meaningful results, such as clinical trials. Moreover, the results found are similar to other reviews conducted for the same purpose. 

### Conclusions

This review suggests that CSS have some benefits for patients with COVID-19. However, the variation in design of the reviewed studies means that there are no comparative results. Further experimental research on CSS and OSS used on patients is required. This review is the first summary of the indicators of relevance to this area of practice and offers future researchers the outcome measures for comparing CSS and OSS. The first step is to establish a protocol study and evidence-based practical guidelines for COVID-19 patients. In order to carry out future experimental studies, we need to unify or specify the criteria in order to understand their possible influences. Key factors include the baseline situation of the patient, standardized criteria for VAP, or diagnosis at admission to the ICU. Furthermore, with endotracheal aspiration, this study shows that we must take into account the specific nursing care procedure adopted because it can be a very influential factor in the outcomes for CSS versus OSS.

## Abbreviations

ABP: arterial blood pressure
CIBIR: Biomedical Research Centre of La Rioja
CSS: closed suction system
ETT: endotracheal tube
GISOSS: Health System Sustainability Research Unit
GRUPAC: Research group in Healthcare
HR: heart rate
ICU: intensive care unit
MV: mechanical ventilation
OR: odds ratio
OSS: open suction system
PEEP: positive and end-expiratory pressure
PIP: peak inspiratory pressure
PRISMA: Preferred Reporting Items for Systematic Reviews and Meta-Analyses
Ri: inspiratory resistance
RR: risk ratio
SpO2: peripheral oxygen saturation
VAP: ventilator-associated pneumonia
WMD: weighted mean difference
